# Neurodevelopment in Early Childhood Affected by Prenatal Lead Exposure and Iron Intake: Erratum

**DOI:** 10.1097/01.md.0000481800.63507.7f

**Published:** 2016-03-11

In the article “Neurodevelopment in Early Childhood Affected by Prenatal Lead Exposure and Iron Intake”,^1^ which appeared in Volume 95, Issue 4 of *Medicine*, Dr. Young Ju Kim's name originally appeared incorrectly. The article has since been corrected online.
